# Functional Analysis of Developmentally Regulated Genes *chs7* and *sec22* in the Ascomycete *Sordaria macrospora*

**DOI:** 10.1534/g3.115.017681

**Published:** 2015-04-14

**Authors:** Stefanie Traeger, Minou Nowrousian

**Affiliations:** Lehrstuhl für Allgemeine und Molekulare Botanik, Ruhr-Universität Bochum, 44780 Bochum, Germany

**Keywords:** sexual development, fruiting body, chitin synthase, SNARE protein, *Sordaria macrospora*

## Abstract

During sexual development, filamentous ascomycetes form complex, three-dimensional fruiting bodies for the generation and dispersal of spores. In previous studies, we identified genes with evolutionary conserved expression patterns during fruiting body formation in several fungal species. Here, we present the functional analysis of two developmentally up-regulated genes, *chs7* and *sec22*, in the ascomycete *Sordaria macrospora*. The genes encode a class VII (division III) chitin synthase and a soluble *N*-ethylmaleimide-sensitive-factor attachment protein receptor (SNARE) protein, respectively. Deletion mutants of *chs7* had normal vegetative growth and were fully fertile but showed sensitivity toward cell wall stress. Deletion of *sec22* resulted in a reduced number of ascospores and in defects in ascospore pigmentation and germination, whereas vegetative growth was normal in the mutant. A SEC22-EGFP fusion construct under control of the native *sec22* promoter and terminator regions was expressed during different stages of sexual development. Expression of several development-related genes was deregulated in the *sec2*2 mutant, including three genes involved in melanin biosynthesis. Our data indicate that *chs7* is dispensable for fruiting body formation in *S. macrospora*, whereas *sec22* is required for ascospore maturation and germination and thus involved in late stages of sexual development.

Sexual development in filamentous ascomycetes involves the differentiation of fruiting bodies containing a number of specialized cell types that are not present in the vegetative mycelium (Bistis *et al.* 2003; Lord and Read 2011). During the last decades, much progress has been made in identifying the mechanisms that control this differentiation process at the molecular level, but a unified model has yet to emerge (Pöggeler *et al.* 2006a). The morphologic changes that occur during fruiting body formation are accompanied by drastic changes in gene expression that are thought to orchestrate the spatiotemporal sequence of events that finally lead to the differentiation and dispersal of ascospores. Not all genes that are differentially expressed during fruiting body development, however, are actually essential for this process, and one way to identify genes that might play an important role in differentiation is the analysis of evolutionary conserved expression patterns, because conserved expression can be a strong indicator of functional significance (Brawand *et al.* 2011; Lehr *et al.* 2014; Nowrousian 2014; Romero *et al.* 2012; Stuart *et al.* 2003). In previous studies, we have compared gene expression during fruiting body development in *Sordaria macrospora* and several other ascomycetes, namely *Neurospora crassa* (Gesing *et al.* 2013; Nowrousian 2009), *Fusarium graminearum* (Gesing *et al.* 2012), and *Pyronema confluens* (Nowrousian and Kück 2006; Traeger *et al.* 2013). In subsequent functional studies, a number of genes with conserved up-regulated expression during fruiting body formation were found to be involved in this process, indicating that conservation of expression is a suitable criterion to select candidate genes for downstream analyses (Gesing *et al.* 2013; Nowrousian 2009; Schindler and Nowrousian 2014).

Here, we present the functional analysis of two additional genes, *chs7* and *sec22*, that were found previously to be up-regulated during sexual development in several fungi (Gesing *et al.* 2012, 2013). The role of both genes during fruiting body differentiation was analyzed in the ascomycete *S. macrospora*, a model organism for the study of sexual development (Engh *et al.* 2010; Teichert *et al.* 2014). *chs7* encodes a chitin synthase (CHS), and *sec22* encodes a SNARE (soluble *N*-ethylmaleimide-sensitive-factor attachment protein receptor) protein. CHS are important proteins for cell-wall biosynthesis and hyphal morphogenesis in fungi. They mediate polymerization of chitin, a major structural component of the fungal cell wall, from the monomer *N*-acetyl-glucosamine (Riquelme and Bartnicki-García 2008; Roncero 2002). Ascomycetes encode up to nine chitin synthase (*chs*) genes in their genomes, with *Schizosaccharomyces pombe* containing only one, the Saccharomycetes three to seven, and filamentous Ascomycetes four to nine genes (Pacheco-Arjona and Ramirez-Prado 2014; Riquelme and Bartnicki-García 2008). Studies of *chs* genes in several ascomycetes have indicated different roles for individual CHS; thus, the expansion of *chs* genes in filamentous ascomycetes might reflect specific needs for chitin biosynthesis capacities during different phases of the life cycle of the respective species (Kong *et al.* 2012; Martín-Udíroz *et al.* 2004; Roncero 2002; Sheng *et al.* 2013). The *S. macrospora chs7* (*SMAC_01722*) gene is orthologous to the *N. crassa chs-6* (*NCU05268*) gene, and both are strongly up-regulated during sexual development in these fungi; in addition, the *S. macrospora chs7* gene is down-regulated in the sterile developmental mutant pro1 (Gesing *et al.* 2013; Teichert *et al.* 2012). Furthermore, a *N. crassa chs-6* deletion mutant showed delayed sexual development and reduced sporulation as a female partner in crosses (Gesing *et al.* 2013); therefore, we chose *chs7* as a candidate for functional analysis in *S. macrospora*.

The second gene that was analyzed is the putative SNARE protein-encoding *sec22* (*SMAC_06625*). SNARE proteins are membrane-associated proteins that are required for intracellular membrane fusions that occur during vesicle trafficking between organelles or to and from the cell surface (Lee *et al.* 2004; Rossi *et al.* 2004; Ungar and Hughson 2003). Sec22p in the yeast *Saccharomyces cerevisiae* is involved in anterograde and retrograde trafficking of vesicles between the endoplasmic reticulum (ER) and the Golgi (Lewis *et al.* 1997; Liu and Barlowe 2002; Spang and Shekman 1998) and was shown to be important in autophagy and the selective accumulation of caesium ions (Dräxl *et al.* 2012; Nair *et al.* 2011). The role of *sec22* homologs during fruiting body development has not been studied yet; however, formation of fruiting bodies requires transport of large amounts of nutrients from the vegetative mycelium to the developing fruiting bodies (Pöggeler *et al.* 2006a); therefore, a role for genes involved in vesicle trafficking might be envisioned. *sec22* was found to be transcriptionally up-regulated during sexual development in *S. macrospora* and *F. graminearum* (Gesing *et al.* 2012; Qi *et al.* 2006) and was thus included in our study.

## Materials and Methods

### Strains and culture conditions

*S. macrospora* strains used in this study are given in Table 1. Unless stated otherwise, standard growth conditions and transformation protocols for *S. macrospora* were as described (Dirschnabel *et al.* 2014; Esser 1982; Nowrousian *et al.* 1999). For the analysis of sexual development, *S. macrospora* was grown on complete medium biomalt maize medium (BMM) (Esser 1982) or minimal medium Sordaria Westergaards medium (SWG) (Nowrousian *et al.* 2005). For stress-tolerance tests, *S. macrospora* was grown on SWG with stated stressors that were supplemented to the medium after sterilization. Growth radius was measured after 2 d under standard growth conditions or different temperatures. Mean and SDs were calculated from three technical replicates for each independent biological replicate. For RNA from cultures developing fruiting bodies, *S. macrospora* was grown at 25° in SWG in surface cultures as described (Nowrousian *et al.* 2005).

**Table 1:** *Sordaria macrospora* strains used in this study

Strain	Genotype	Reference
Wild type (S91327)	Wild type	Strain collection*^a^*
fus1-1 (S84595)	Spore color mutant	(Nowrousian *et al.* 2012)
∆ku70 (S96888)	∆ku70	(Pöggeler and Kück 2006)
∆sec22 (S121397)	∆sec22	This study
SEC22-GFP-NA (ST13.1)	∆sec22::P*sec22*::*sec22-gfp*	This study
SEC22-GFP-OE (S119860)	∆sec22::P*gpd*::*sec22-gfp*	This study
∆chs7-1 (S123091)	∆chs7-1	This study
∆chs7-2 (S123107)	∆chs7-2	This study
∆chs7-3 (S123114)	∆chs7-3	This study
∆chs7-4 (S123147)	∆chs7-4	This study

All strains are single ascospore isolates.

a Strain collection at the Department of General and Molecular Botany, Bochum.

### Preparation of nucleic acids and reverse-transcription quantitative polymerase chain reaction (RT-qPCR)

DNA and RNA were extracted using standard protocols (Pöggeler *et al.* 1997; Yarden *et al.* 1992). RT-qPCR was performed as described previously (Schindler and Nowrousian 2014), oligonucleotides used as primers are given in Supporting Information, Table S1.

### Generation of ∆chs7 mutants

For the generation of plasmids and homologous recombination in *S. cerevisiae* PJ69-4a, standard procedures were used (Bloemendal *et al.* 2012; Colot *et al.* 2006; James *et al.* 1996; Sambrooke and Russell 2001). For the *chs7* knockout construct, the flanking regions of *chs7* (*SMAC_01722*) were amplified with specific oligonucleotides [5′ region 1722-5-fw/rv (965 bp), 3′ region 1722-3-fw/rv (967 bp), Table S1] using PCR based on wild-type genomic DNA; the Hygromycin resistance cassette was obtained from vector pDrivehph (pSF27-34) (Nowrousian and Cebula 2005) by *Eco*RI digestion; and all fragments were inserted into pRS426 (Christianson *et al.* 1992) by homologous recombination in yeast. The resulting plasmid p∆chs7 was used as template to amplify the 3.4-kb knockout cassette with primers 1722-5-fw/-3-rv in a Phusion DNA Polymerase (Thermo Scientific)-PCR. The knockout cassette was used to transform *S. macrospora* strain Δku70 (Pöggeler and Kück 2006). Primary transformants were crossed against the fus1-1 strain to generate ∆chs7 strains without the Δku70 background, and transformants were screened for homologous integration by PCR and Southern blot analysis as described previously (Nowrousian and Cebula 2005) (Figure S1). For further analyses four strains (∆chs7-1: S123091; ∆chs7-2: S123107; ∆chs7-3: S123114; ∆chs7-4: S123147) were chosen.

### Generation of ∆sec22 mutants and complementation

Generation of the *sec22* deletion construct p∆sec22 was performed as described previously by the use of oligonucleotides 6625-5-fw/rv and 6625-3-fw/rv (Table S1) to amplify fragments of 1 kb upstream and 0.5 kb downstream of the *sec22* open reading frame. The 3.4-kb knockout cassette was amplified from the resulting plasmid p∆sec22 with oligonucleotides 6625-5-fw/-3-rv and used to transform *S. macrospora* strain Δku70. Transformants were screened for homologous integration by PCR and Southern blot analysis as described previously (Nowrousian and Cebula 2005). Primary transformants were crossed against the fus1-1 strain to generate ∆sec22 strains without the Δku70 background, and transformants were screened for homologous integration by PCR and Southern blot analysis as described previously (Nowrousian and Cebula 2005).We were not able to isolate a single spore without the *sec22* gene still detectable via PCR. Therefore we decided to follow a sheltered rescue plan to investigate if *sec22* is an essential gene for *S. macrospora*. Overexpression construct pSEC22-GFP-OE with *sec22*::*egfp* under the control of the *Aspergillus nidulans gpd* promotor was cloned and ectopically integrated into the wild-type strain. An ascospore isolate with one copy of the overexpression plasmid was used for further crosses with transformants carrying the knockout construct at the homologous locus to establish a transformant carrying both the deletion background of *sec22* and an ectopically integrated overexpression construct. One of these strains (S119860) was crossed against the fus1-1 strain to obtain three ∆sec22 strains, which were confirmed as deletion strains via PCR and Southern blot analysis (Figure S2). For complementation, plasmid pSEC22-GFP-NA was cloned with *sec22*::*egfp* under the native *sec22* promotor and terminator. Plasmid pSEC22-GFP-NA was transformed in ∆sec22 (S121397), and single spore isolate SEC22-GFP-NA (ST13.1) was used for further analyses.

### Microscopic analyses

To investigate differentiation, *S. macrospora* strains were grown on slides with 1 mL of BMM medium at 27° in continuous light (Engh *et al.* 2007b). Hyphal fusion assays were performed as described previously (Bloemendal *et al.* 2012). Light and fluorescence microscopy were performed as described previously (Dirschnabel *et al.* 2014; Engh *et al.* 2007b).

### Ascospore quantification analyses

Germination analyses of ascospores were performed as described previously (ascospore germination assay of selfing strains) (Dirschnabel *et al.* 2014). For quantification of released ascospores and their pigmentation, Petri dish lids of three technical replicates per independent biological replicate grown on BMM were analyzed. Ascospores were washed off with distilled water, counted by hematocytometer, and analyzed for pigmentation.

## Results

### CHS7 belongs to division III of CHS

**Figure 1:**
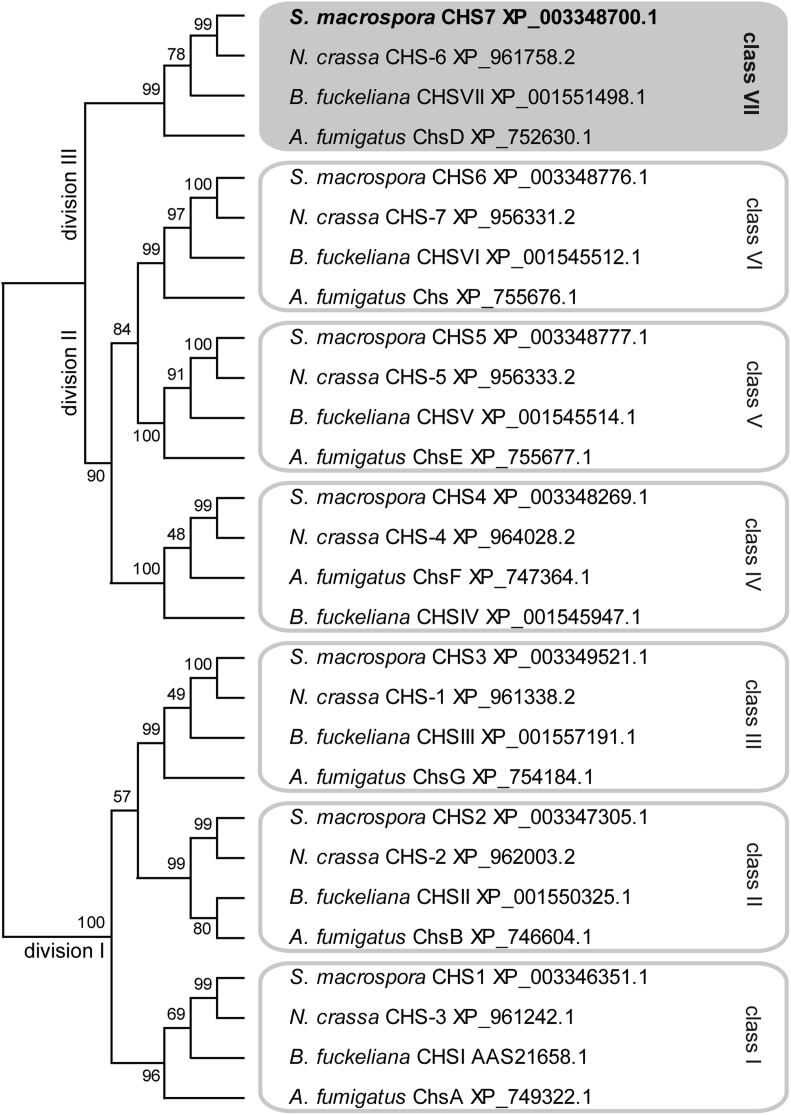
Phylogenetic analysis of chitin synthases from four ascomycetes. A multiple alignments was created with CLUSTALW (Thompson *et al.* 2002) and used for maximum likelihood analysis with MEGA5.10 (Tamura *et al.* 2011). Numbers at branches indicate bootstrap support in % for 1000 bootstrap replications. Classes and divisions are labeled according to Choquer *et al.* (2004) and Riquelme and Bartnicki-García (2008), respectively. The predicted *S. macrospora* Chitin synthases (CHS) proteins are encoded by genes with the following locus tag numbers in the genome sequence (Nowrousian *et al.* 2010): CHS1, *SMAC_07828*; CHS2, *SMAC_07162*; CHS3, *SMAC_03109*; CHS4, *SMAC_02767*; CHS5, *SMAC_01800*; CHS6, *SMAC_01799*; CHS7, *SMAC_01722*.

To characterize the predicted CHS SMAC_01722 and other CHS in *S. macrospora*, we used BLAST searches (Altschul *et al.* 1997) in the *S. macrospora* predicted peptides and identified seven putative CHS that are orthologous to the seven *N. crassa* CHS (Borkovich *et al.* 2004; Riquelme and Bartnicki-García 2008) (Figure 1). CHS in ascomycetes can be divided into seven classes according to sequence similarity and domain structure. Two nomenclature systems are in use that are similar for classes I to V but differ in that classes VI and VII are exchanged (Choquer *et al.* 2004; Mandel *et al.* 2006), going back to the assignment of class VI to two different types of CHS at almost the same time (Chigira *et al.* 2002; Roncero 2002). In this study, we use the nomenclature by Choquer *et al.* (2004), where class VI consists of CHS with a myosin-like domain that is not present in class VII CHS. Classes of CHS can be grouped into divisions by phylogenetic analysis, with division I consisting of classes I, II, and III, which are characterized by an additional catalytic subdomain pfam08407 (Fajardo-Somera *et al.* 2015; Riquelme and Bartnicki-García 2008). Division II contains the classes IV, V, and VI, which carry a cytochrome b5-like domain, with classes V and VI carrying an additional myosin-head-like domain (Choquer *et al.* 2004). Division III consists of class VII CHS that have the simplest domain structure and carry no other domains besides the conserved CHS catalytic domain (pfam03142) present in all CHS (Choquer *et al.* 2004; Riquelme and Bartnicki-García 2008). A phylogenetic analysis showed that the seven *S. macrospora* CHS represent the seven CHS classes, similar to *N. crassa* and several other filamentous ascomycetes (Figure 1). The developmentally upregulated *SMAC_01722* (XP_003348700.1) encodes a CHS that belongs to class VII, division III; therefore, the gene was named *chs7*.

### A *chs7* deletion mutant is fertile but sensitive to cell-wall stress

**Figure 2:**
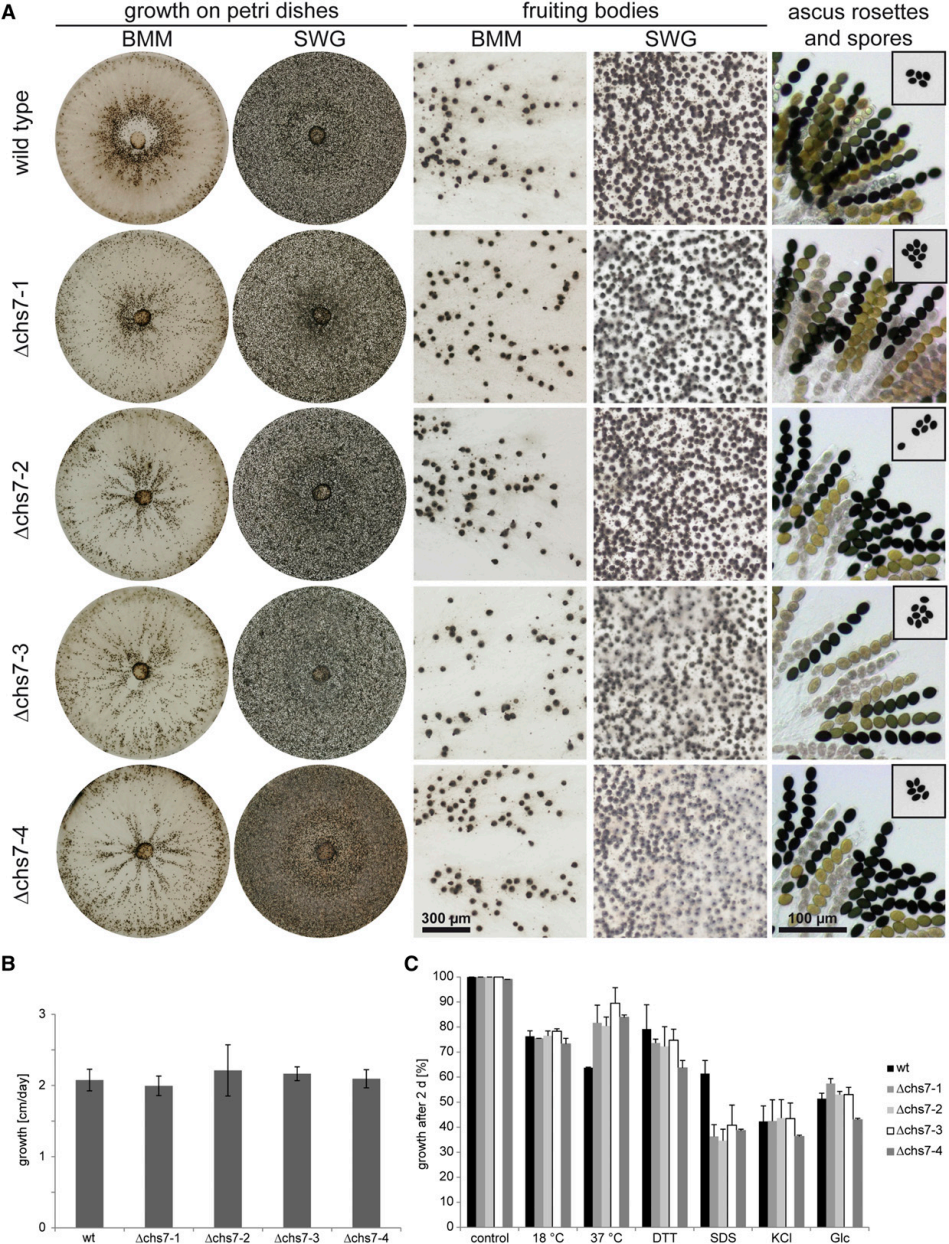
Analysis of sexual development, vegetative growth, and stress resistance in Δchs7 strains. (A) Sexual development of the wild-type and four Δchs7 strains after 7 d on complete medium (BMM) and minimal medium (SWG). All strains form perithecia and ascus rosettes (right column), and eject ascospores toward the lids of the Petri dishes (spores on lids shown in small boxes in right column). (B) Vegetative growth was analyzed on BMM in race tubes over 10 d, error bars give SDs of three independent biological replicates. (C) Stress resistance was analyzed on SWG in Petri dishes with the indicated stress-inducing agents at the following concentrations: dithiothreitol (DTT) 0.04%, sodium dodecyl sulfate (SDS) 0.1%, KCl 0.6 M, glucose 0.6 M. The wild type was used as reference for the control. For each stress-inducing condition, the growth on the control plates for the corresponding strain was used as reference. Error bars indicate SDs for two independent biological replicates, each with three technical replicates.

To test whether *chs7* is involved in fruiting body formation in *S. macrospora*, we generated deletion mutants by homologous recombination (Figure S1). Four Δchs7 strains (Δchs7-1 to Δchs7-4) were analyzed for phenotypes related to sexual development, but all were fully fertile and produced perithecia, asci and ascospores in similar numbers as the wild type (Figure 2A). The growth rate of the vegetative mycelium also was unaffected in the Δchs7 mutants (Figure 2B). Chitin is an important structural component of the fungal cell wall, and CHS mutants in several fungi were reported to be sensitive to stress conditions, including cell wall stressors or osmotic stress (Kong *et al.* 2012; Martín-Udíroz *et al.* 2004; Sheng *et al.* 2013). Therefore, we tested growth of the Δchs7 strains under different stress conditions (Figure 2C). At 18°, growth of all strains was reduced compared to the standard growth temperature of 25°, but the reduction was similar to that of the wild type. Somewhat surprisingly, growth at 37° is less affected in the Δchs7 strains than in the wild type, indicating a greater heat stress tolerance in the mutant strains. Osmotic stress induced by KCl or glucose led to a similar growth reduction in the wild-type and the mutant strains, and the same was observed for the ER stress-inducing agent dithiothreitol (DTT); however, cell-wall stress induced by sodium dodecyl sulfate (SDS) in the medium led to much stronger growth reduction in the four Δchs7 deletion strains than in the wild type (Figure 2C). This finding might indicate that the Δchs7 strains have a reduced chitin content or modified cell-wall composition, as was described for mutants in the *Aspergillus fumigatus* homolog *chsD* (Mellado *et al.* 1996).

### A *sec22* deletion mutant has defects in ascospore maturation and germination

**Figure 3:**
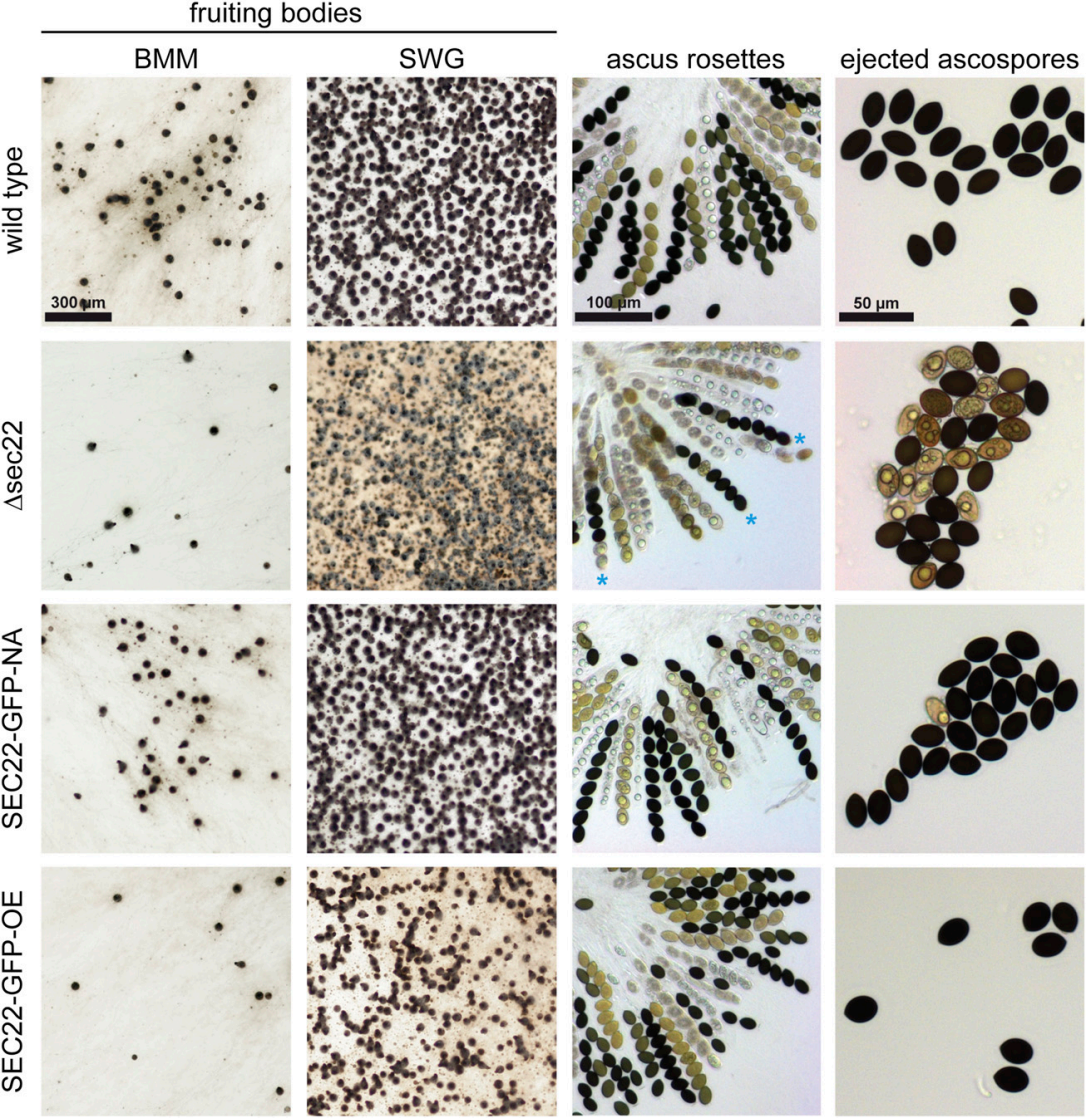
Sexual development in the Δsec22 mutant and complemented strains. Strain SEC22-GFP-NA expresses *sec22* under its own promoter and terminator regions, whereas in SEC22-GFP-OE *sec22* is under control of the strong, constitutive *A. nidulans gpd* promoter. Fruiting body formation was analyzed after 7 d on complete medium (BMM) and minimal medium (SWG). Ascus rosettes from perithecia grown on BMM medium were analyzed after 7−9 d. Ascospores from the Δsec22 strain show different degrees of melanization within one ascus (blue asterisks). Ascospores that were ejected toward the lid of the plate from strains grown on BMM were analyzed after 7−9 d (right column).

**Figure 4:**
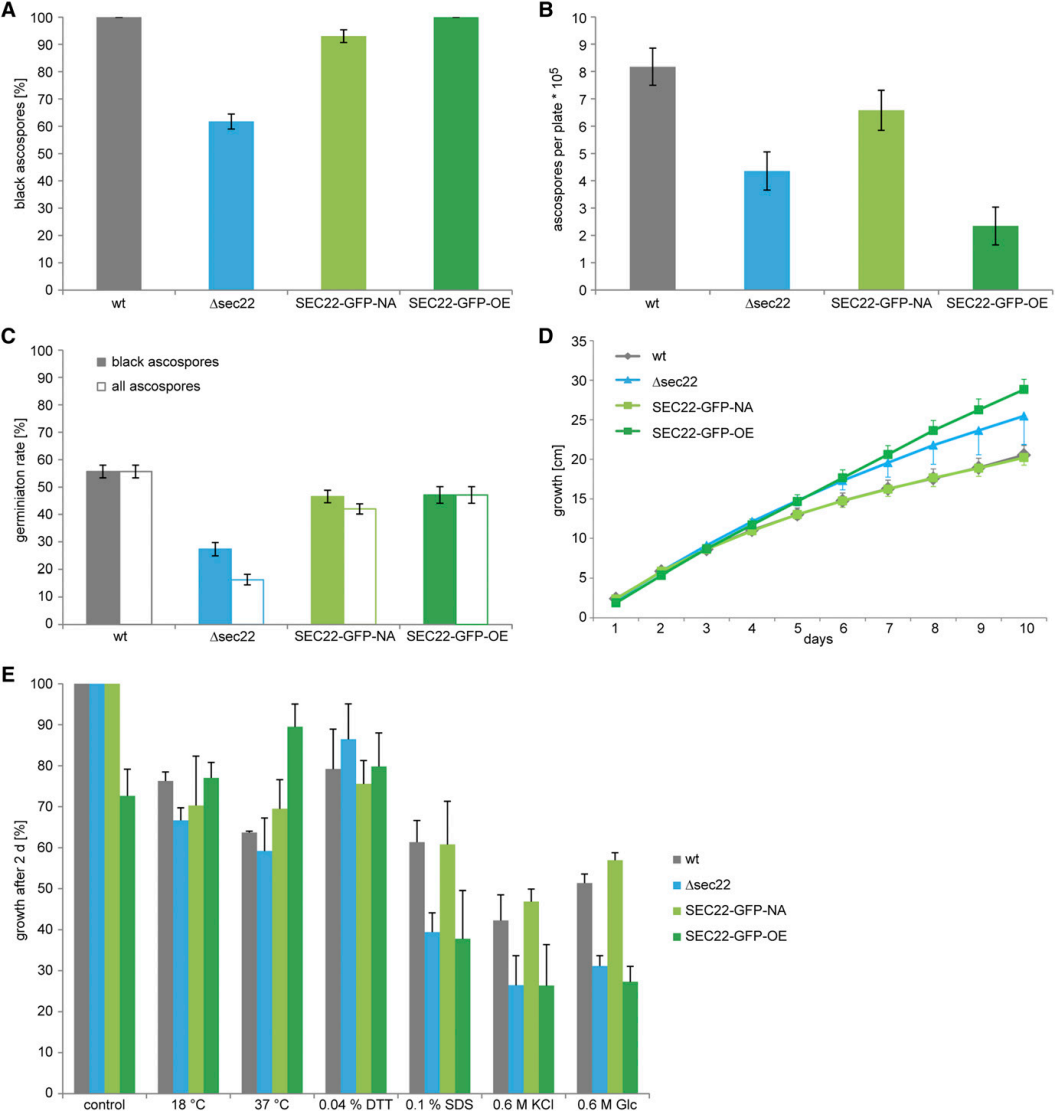
Analysis of ascospore production and germination, and stress resistance in the Δsec22 mutant and complemented strains. Strains are the same as in Figure 3. (A) Proportion of black ascospores that are ejected from perithecia. Error bars give standard deviations for three independent biological replicates. (B) Number of ejected ascospores per Petri dish. Error bars give SDs for three independent biological replicates. (C) Germination rate of ascospores. The wild type and strain SEC22-GFP-OE eject only black spores; therefore, the germination rate is identical for black and all ascospores. Strains Δsec22 and SEC-22-NA eject a certain proportion of nonmelanized ascospores (A), none of which were observed to germinate, thereby reducing the germination rate of all ascospores. Error bars give standard deviations of three independent biological replicates. (D) Vegetative growth was analyzed on race tubes over 10 d. Error bars give SDs of three independent biological replicates, for clarity, only one direction (plus or minus) is shown for each error bar. (E) Stress resistance was analyzed on SWG in petri dishes with the indicated stress-inducing agents at the following concentrations: dithiothreitol (DTT) 0.04%, sodium dodecyl sulfate (SDS) 0.1%, KCl 0.6 M, glucose 0.6 M. The wild type was used as reference for the control. For each stress-inducing condition, the growth on the control plates for the corresponding strain was used as reference. Error bars indicate SDs for two independent biological replicates, each with three technical replicates.

The developmentally up-regulated *S. macrospora sec22* (*SMAC_06625*, XP_003346158.1) encodes a conserved protein with the typical domain structure described for this class of SNARE proteins (Rossi *et al.* 2004; Ungar and Hughson 2003) (Figure S3). To test whether *sec22* plays a role in sexual development, we generated a *sec22* deletion mutant (Figure S2). The mutant forms normal fruiting bodies and eight-spored asci; however, ascospore pigmentation is different from the wild type (Figure 3). In the wild type, the eight ascospores within an ascus usually display the same degree of maturation, which can be seen in the degree of pigmentation from immature, nonpigmented to mature, completely melanized spores. In the mutant strain, spores within an ascus frequently show different degrees of pigmentation, *i.e.*, light-brown and fully melanized spores reside within one ascus (Figure 3). Furthermore, the mutant ejects ascospores that are not fully mature, in contrast to the wild type, where only fully melanized ascospores are released from the perithecia (Figure 3). Quantification of ascospores showed that the mutant ejects only about 60% of black ascospores compared to the wild type (Figure 4A), and that the overall number of ejected ascospores is also reduced by nearly 50% (Figure 4B). Furthermore, the germination rate of the ascospores from the Δsec22 mutant is reduced. Only the fully melanized ascospores from the mutant were able to germinate, whereas none of the not fully pigmented spores germinated. However, even the fully melanized ascospores from the mutant showed a lower germination rate than those of the wild type (Figure 4C), indicating other spore maturation or germination defects besides pigmentation.

**Figure 5:**
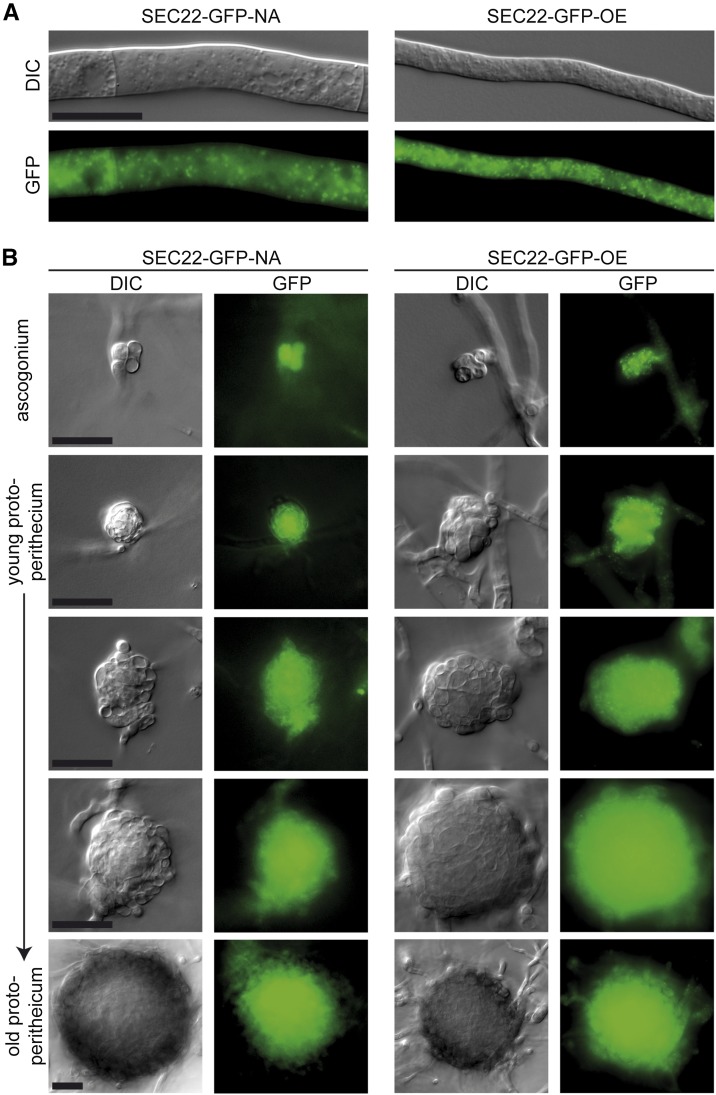
Microscopic analysis of SEC22-EGFP fusion proteins shows that *sec22* is expressed throughout the developmental cycle. Strain SEC22-GFP-NA expresses *sec22* under its own promoter and terminator regions, whereas in SEC22-GFP-OE *sec22* is under control of the strong, constitutive *A. nidulans gpd* promoter. EGFP fluorescence was observed in vegetative hyphae (A) and developing ascogonia and protoperithecia (B). Scale bars indicate 20 µm.

**Figure 6:**
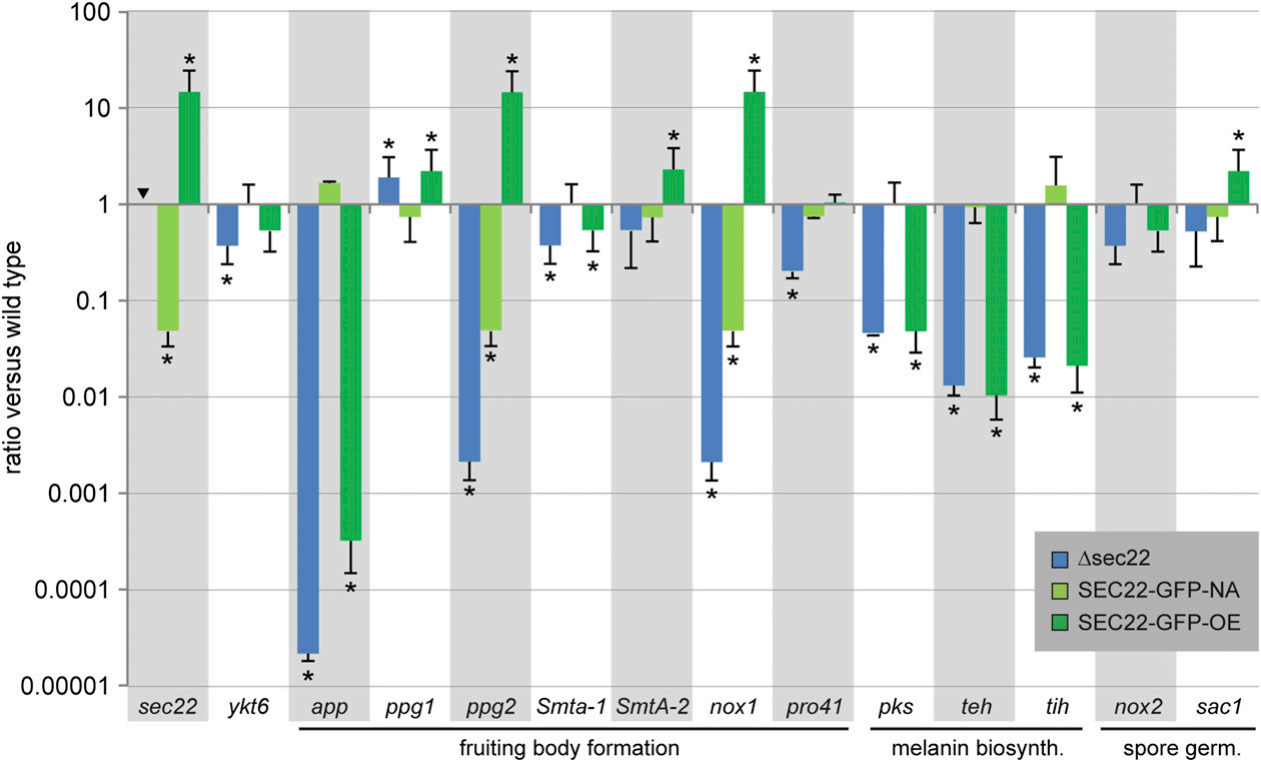
Expression of developmentally regulated genes in the Δsec22 mutant and complemented strains. Strains are the same as in Figure 3. Transcript levels determined by RT-qPCR are given relative to the wild type; the mean of two independent biological replicates is shown. Error bars indicate SD (positive values; for better visualization standard deviations for negative expression ratios are shown in the negative direction). For *sec22*, no expression was detected in the *sec22* deletion mutant as expected (indicated by an inverted triangle). Asterisks indicate significantly differential expression (*P* ≤ 0.001, calculated with REST) (Pfaffl *et al.* 2002).

Two complementation plasmids were constructed for *sec22*, one expressing a *sec22-egfp* fusion gene under its own promoter and terminator, and the other an overexpression construct with *sec22-egfp* under control of a constitutive promoter. Both constructs led to EGFP fluorescence in dots within the cytoplasm of hyphae of complemented strains (Figure 5A), and fluorescence in developing fruiting bodies was strong with both constructs (Figure 5B). Expression of *sec22* was detectable at transcript level in both strains, with the expression from the overexpression construct stronger, and from the native construct somewhat lower than in the wild type (Figure 6). The spore pigmentation and germination defects of the Δsec22 mutant were complemented in the mutant strain carrying the construct that expresses *sec22* under its own promoter and terminator regions (strain SEC22-GFP-NA, Figure 3 and Figure 4). This strain produces mostly fully pigmented ascospores, and germination of these spores is in the same range as for the wild type. Interestingly, a mutant strain carrying the construct overexpressing *sec22* (strain SEC22-GFP-OE) shows full restoration of ascospore pigmentation and germination, but the number of ascospores that are produced is more similar to the mutant than to the wild type (Figure 3 and Figure 4). One reason for this finding might be a reduced number of fruiting bodies that are produced in BMM medium by the Δsec22 mutant and the overexpressing strain, which is the medium that was used for the ascospore quantification and germination assays (Figure 3).

We also investigated vegetative growth and stress tolerance of Δsec22 and complemented strains. Vegetative growth was similar to the wild type for all strains, with the deletion and overexpression strains showing slightly but not significantly faster growth (Figure 4D). When grown under stress-inducing conditions, the mutant as well as the overexpression strain showed growth reduction under cell-wall stress induced by sodium dodecyl sulfate, and osmotic stress induced by glucose, and to a lesser degree by KCl, whereas the complemented strain SEC22-GFP-NA had wild type-like growth (Figure 4E).

### *sec22* is required for correct expression of several development-associated genes

Because *sec22* is necessary for full fertility, we wondered whether the deletion of *sec22* has an effect on the expression of other genes known to be involved in or associated with sexual development in *S. macrospora*. RT-qPCR was performed for 12 development-associated genes from *S. macrospora* as well as for *ykt6* (*SMAC_01930*), because *ykt6* in *S. cerevisiae* was shown to be upregulated and able to functionally replace *sec22* in a *sec22* mutant strain (Liu and Barlowe 2002). In contrast to *S. cerevisiae*, however, *ykt6* is down-regulated in the Δsec22 mutant of *S. macrospora* (Figure 6), and the ascospore maturation and germination phenotype of the Δsec22 mutant shows that *ykt6* cannot functionally replace *sec22* in *S. macrospora*.

Several genes that are required or associated with fruiting body formation were tested for expression in Δsec22 and the complemented strains (Figure 6). The two mating-type genes *Smta-1* and *SmtA-2* that are essential for fruiting body formation (Klix *et al.* 2010; Pöggeler *et al.* 2006b) are slightly down-regulated in the *sec22* mutant, although only expression of *Smta-1* is significantly different from the wild type. In addition to the mating-type genes, the two pheromone genes *ppg1* and *ppg2* are involved in sexual development (Mayrhofer *et al.* 2006). Interestingly, while *ppg1* is slightly up-regulated, *ppg2* is strongly down-regulated in Δsec22. One might speculate that the different expression patterns of the pheromone genes in Δsec22 are related to the different modes of secretion of the corresponding pheromones. While the *ppg1*-derived pheromone is similar to the *S. cerevisiae* α-factor, and therefore most likely secreted via the classical ER-based secretion pathway, the *ppg2*-derived pheromone is similar to the *S. cerevisiae* **a**-factor, which is secreted by an ATP-binding cassette transporter (Mayrhofer and Pöggeler 2005). Thus, secretion of the PPG1 pheromone might involve a SEC22-dependent pathway, and it might be hypothesized that autoregulatory feedback leads to upregulation of *ppg1* expression when this pathway is not fully functional. Secretion of the PPG2 pheromone is unlikely to involve SEC22, and therefore the down-regulation of *ppg2* is probably a more indirect effect.

The *pro41* gene that encodes an ER membrane protein essential for fruiting body formation (Nowrousian *et al.* 2007a) is also down-regulated in the *sec22* mutant. Interestingly, the PRO41 orthologs BcNoxD and PaNoxD from *Botrytis cinerea* and *Podospora anserina*, respectively, colocalize and/or interact with an NADPH oxidase (NOX) at the ER and vacuolar membranes as part of a NOX complex (Lacaze *et al.* 2015; Siegmund *et al.* 2015). The NOX proteins that are part of this complex are PaNox1 in *P. anserina*, and BcNoxA in *B. cinerea*, which are homologs of NOX1 in *S. macrospora* (Dirschnabel *et al.* 2014; Malagnac *et al.* 2004; Siegmund *et al.* 2013), and *nox1* is also strongly down-regulated in the Δsec22 mutant (Figure 6). Thus, one might hypothesize that genes for ER proteins or proteins that require ER-dependent export are misregulated in Δsec22, probably due to feedback reactions caused by insufficient or de-regulated transport events.

This hypothesis would also explain the down-regulation of the three melanin biosynthesis genes *pks*, *teh*, and *tih* (Figure 6). Melanin is present in the cell wall of many fungal structures, and its intermediates and/or biosynthetic enzymes are thought to require vesicle-based transport (Eisenman and Casadevall 2012). In *S. macrospora*, melanin is required for the black pigmentation of perithecia and ascospores, and the melanin genes are up-regulated during sexual development (Engh *et al.* 2007a; Nowrousian *et al.* 2012). The down-regulation of the melanin biosynthesis genes in Δsec22 correlates well with the ascospore pigmentation defects in the mutant; however, in the overexpression strain SEC22-GFP-OE, the genes are also down-regulated (Figure 6), despite the normal pigmentation in this strain (Figure 3). One possible explanation for this finding might be that the bottleneck of melanin biosynthesis is not the level of transcription of the biosynthesis genes, but ER-related transport processes. If this were the case, transcriptional down-regulation in the Δsec22 strain might be caused by feedback due to ER stress, whereas in the overexpression strain, it might be possible that more efficient transport leads to similar transcriptional down-regulation through product-based inhibition.

One other gene that was tested is the development-associated *app* gene, which encodes a protein that accumulates in fruiting bodies in *S. macrospora* and *N. crassa*. Previously, *app* was found to be down-regulated in mutants with a block at the stage of young fruiting bodies (protoperithecia), but normal in a developmental mutant that produced fruiting bodies, but no mature spores (Nowrousian *et al.* 2007b). Therefore, it was surprising that *app* is strongly down-regulated in the *sec22* mutant, even though the mutant produces fruiting bodies (Figure 6). However, in a recent analysis of a polyketide synthase mutant that only produces protoperithecia, it was found that *app* was expressed normally in this mutant and down-regulated in overexpressing strains, indicating that *app* expression is regulated by at least two different developmental pathways (Schindler and Nowrousian 2014), which could also explain down-regulation of *app* in the fertile *sec22* mutant.

We also analyzed transcript levels of *nox2* and *sac1*, both of which are involved in ascospore germination in *S. macrospora* (Dirschnabel *et al.* 2014; Kamerewerd *et al.* 2008); however, both genes are expressed normally in Δsec22 (Figure 6).

## Discussion

### *chs7* orthologs play different roles during growth and development in different species

In this study, we found that the *S. macrospora chs7* is not required for sexual development but instead plays a role under stress conditions. Interestingly, a deletion mutant of the *N. crassa* ortholog *chs-6* has a very different phenotype. It is strongly reduced in vegetative growth (Fajardo-Somera *et al.* 2015; Sánchez-León *et al.* 2011), in contrast to the *S. macrospora* Δchs7 strain, which grows normally (Figure 2). Furthermore, the *N. crassa* Δchs-6 is delayed in sexual development and sporulation when present as the female partner in a cross and forms fewer perithecia and no ascospores in homozygous crosses (Fajardo-Somera *et al.* 2015; Gesing *et al.* 2013), whereas the *S. macrospora* Δchs7 has wild type-like sexual development (Figure 2). Thus, these orthologous genes have evolved different functions in these relatively closely related species. However, even though orthologs from *S. macrospora* and *N. crassa* often have similar functions, different roles for ortholog pairs in these species have been observed previously. Examples are the orthologous transcription factor genes *pro1* and *adv-1* that are involved in sexual development, but not in vegetative growth, in *S. macrospora*, and in sexual development as well as vegetative growth in *N. crassa* (Colot *et al.* 2006; Masloff *et al.* 1999), and *pro45* and its *N. crassa* ortholog *ham-4*, which are required for sexual development in *S. macrospora* but not *N. crassa* (Nordzieke *et al.* 2015; Simonin *et al.* 2010). In the case of CHS, the presence of multiple paralogs in the genomes of most ascomycetes might facilitate rapid evolution leading to functional differentiation (Pacheco-Arjona and Ramirez-Prado 2014; Riquelme and Bartnicki-García 2008). In *Magnaporthe oryzae*, which is a member of the Sordariomycetes, but only distantly related to *S. macrospora* and *N. crassa*, the *chs7* ortholog is required for the formation of appressoria from germ tubes on hydrophobic surfaces, and for penetration and invasive growth during plant infection (Kong *et al.* 2012). However, sexual development in the corresponding mutant has not yet been studied in *M. oryzae*. In the Eurotiomycete *Aspergillus nidulans*, the *chs7* ortholog *chsG* is not involved in vegetative growth or sexual development (Fajardo-Somera *et al.* 2015), whereas the *A. fumigatus* ortholog *chsD* is required for the full chitin content of the cell wall, but is dispensable for infection in a mouse model of aspergillosis (Mellado *et al.* 1996). Thus, *chs7* orthologs have evolved to fulfill a number of species-specific roles in growth, sexual development, and pathogenicity in different ascomycetes. Nevertheless, several CHS paralogs can be partially redundant and can complement each other in case of deletion of one paralog. For example, *N. crassa* single mutants of *chs-1* and *chs-3* have normal vegetative growth, whereas the double mutant shows severe growth reduction (Fajardo-Somera *et al.* 2015; Sánchez-León *et al.* 2011), and in *M. oryzae*, *chs5* can partially complement the function of *chs6* (Kong *et al.* 2012). However, these functional redundancies occur for CHS that are members of the same division, with *N. crassa chs-1* and *chs-3* being members of division I, and *M. oryzae chs5* and *chs6* members of division II (Kong *et al.* 2012; Riquelme and Bartnicki-García 2008). The *S. macrospora chs7* is the only member of division III; however, CHS from division III have the simplest domain structure without additional domains apart from the essential CHS domain (Fajardo-Somera *et al.* 2015), therefore functional replacement by other CHS might seem possible, which could explain the lack of a developmental phenotype despite a conserved expression pattern.

### The SNARE protein SEC22 is involved in late stages of sexual development

The functional analysis of *sec22* showed that this gene is required for normal ascospore maturation and germination in *S. macrospora*. To the best of our knowledge, this is the first time that an involvement of *sec22* in sexual development in fungi is shown. In *S. cerevisiae*, *sec22* mutants are sensitive to heat stress, but grow normally under standard laboratory conditions, because of functional redundancy with the SNARE-encoding *ykt6* gene, whereas a double mutant of both genes is lethal (Dascher *et al.* 1991; Liu and Barlowe 2002). The *S. macrospora sec22* mutant is not heat sensitive, but overexpression of *sec22* leads to increased vegetative growth at higher temperature (Figure 4E). In filamentous fungi, *sec22* was analyzed in *M. oryzae*, where it was found to be involved in conidiation, pathogenicity, and growth; furthermore, the corresponding mutant is more sensitive to oxidative stress (Song *et al.* 2010). Sexual development or the reaction to heat stress was not analyzed in the *M. oryzae* mutant yet. Interestingly, SEC22 in *Arabidopsis thaliana* is essential for male and female gametophyte development, and thus for the completion of the developmental cycle of this plant (El-Kasmi *et al.* 2011). The findings in the filamentous fungi *M. oryzae* and *S. macrospora* and the plant *A. thaliana* indicate that *sec22* might have species-specific roles in multicellular development.

In *S. cerevisiae*, *sec22* was shown to be involved in macroautophagy, a process that mediates intracellular degradation of proteins and organelles through the formation of autophagosome vesicles. Interestingly, this requirement for *sec22* in yeast is not compensated by *ykt6* as described for other functions of *sec22* (Nair *et al.* 2011). Autophagy is conserved in all eukaryotes, and in *S. macrospora*, several autophagy genes are essential for sexual development (Teichert *et al.* 2014). Among these are the genes *Smatg4* and *Smatg8*. Mutants for both genes are unable to form mature fruiting bodies, and display reduced ascospore germination in crosses against the wild type or each other (Voigt and Pöggeler 2013). The latter phenotype is similar to the reduced spore germination in Δsec22, and therefore one might hypothesize that *sec22* is also involved in autophagy in *S. macrospora*. However, any role of *sec22* in autophagy in *S. macrospora* is unlikely to be an essential one, because mutants in core autophagy genes that were generated so far are either nonviable or unable to generate fruiting bodies (Nolting *et al.* 2009; Voigt *et al.* 2013; Voigt *et al.* 2014; Voigt and Pöggeler 2013), whereas the Δsec22 phenotype concerns ascospore maturation and germination, and thus occurs during later stages of sexual development.

Our analysis revealed transcriptional deregulation of several developmental genes in the Δsec22 mutant (Figure 6). Several of these genes encode ER-associated proteins or proteins that are predicted to require vesicle-dependent transport steps, suggesting that their deregulation might be caused by feedback mechanisms that sense problems in the vesicle trafficking pathway. The hyphae of filamentous fungi are extremely polarized cells that require a continuous flow of vesicles to the hyphal tips to support polarized growth (Fischer *et al.* 2008; Sánchez-León *et al.* 2015), and the differentiation of fruiting bodies and spores in particular requires the transfer of large amounts of metabolites within the fungal mycelium (Pöggeler *et al.* 2006a; Wessels 1993). It is conceivable that *sec22* might be involved in the trafficking processes required for ascospore maturation. Highly specific roles for *sec22* in transport processes can also be found in other species, *e.g.*, it is essential for accumulation of caesium ions (Cs^+^), but not for accumulation of the essential K^+^ ions in the vacuoles of *S. cerevisiae* and *A. thaliana* (Dräxl *et al.* 2012). Thus, the molecular function of the SNARE protein SEC22 might be conserved, while integration into cellular and organismic processes appears to be variable among eukaryotes.

## Supplementary Material

Supporting Information
