# Supplementary material for: Functional Analysis of Developmentally Regulated Genes chs7 and sec22 in the Ascomycete Sordaria macrospora
Source: G3 (Bethesda). 2015 Apr 14;5(6):1233–45. doi: 10.1534/g3.115.017681 (PMC4478551; doi:10.1534/g3.115.017681)
Supplement: Supporting Information [file supp_5_6_1233__index.html]

Functional Analysis of Developmentally Regulated Genes chs7 and sec22 in the Ascomycete Sordaria macrospora — Supporting Information 

# Functional Analysis of Developmentally Regulated Genes *chs7* and *sec22* in the Ascomycete *Sordaria macrospora*

## Supporting Information for Traeger and Nowrousian, 2015

**Files in this Data Supplement:**

- Supporting Information - Figures S1-S3 and Table S1 (PDF, 924 KB)
- Figure S1 - Deletion of *S. macrospora chs7*. (PDF, 203 KB)
- Figure S2 - Deletion of *S. macrospora sec22*. (PDF, 203 KB)
- Figure S3 - Multiple alignment of SEC22 homologs from four ascomycetes. (PDF, 274 KB)
- Table S1 - Oligonucleotides used in the study. (PDF, 403 KB)
