# Supplementary material for: Functional Analysis of Developmentally Regulated Genes chs7 and sec22 in the Ascomycete Sordaria macrospora
Source: G3 (Bethesda). 2015 Apr 14;5(6):1233–45. doi: 10.1534/g3.115.017681 (PMC4478551; doi:10.1534/g3.115.017681)
Supplement: Supporting Information [file supp_g3.115.017681_017681SI.pdf]

G3 · Genes | Genomes | Genetics

Functional analysis of developmentally regulated genes *chs7* and *sec22* in the ascomycete *Sordaria macrospora*

Stefanie Traeger and Minou Nowrousian*

Lehrstuhl für Allgemeine und Molekulare Botanik, Ruhr-Universität Bochum, 44780 Bochum, Germany

*corresponding author:
Minou Nowrousian, Ph.D., Lehrstuhl für Allgemeine und Molekulare Botanik, Fakultät für Biologie und Biotechnologie, Ruhr-Universität Bochum, Universitätsstr. 150, 44780 Bochum, Germany, phone: +49 234 3224588, email: minou.nowrousian@rub.de

Supporting Figures 1-3
Supporting Table 1

DOI: 10.1534/g3.115.017681

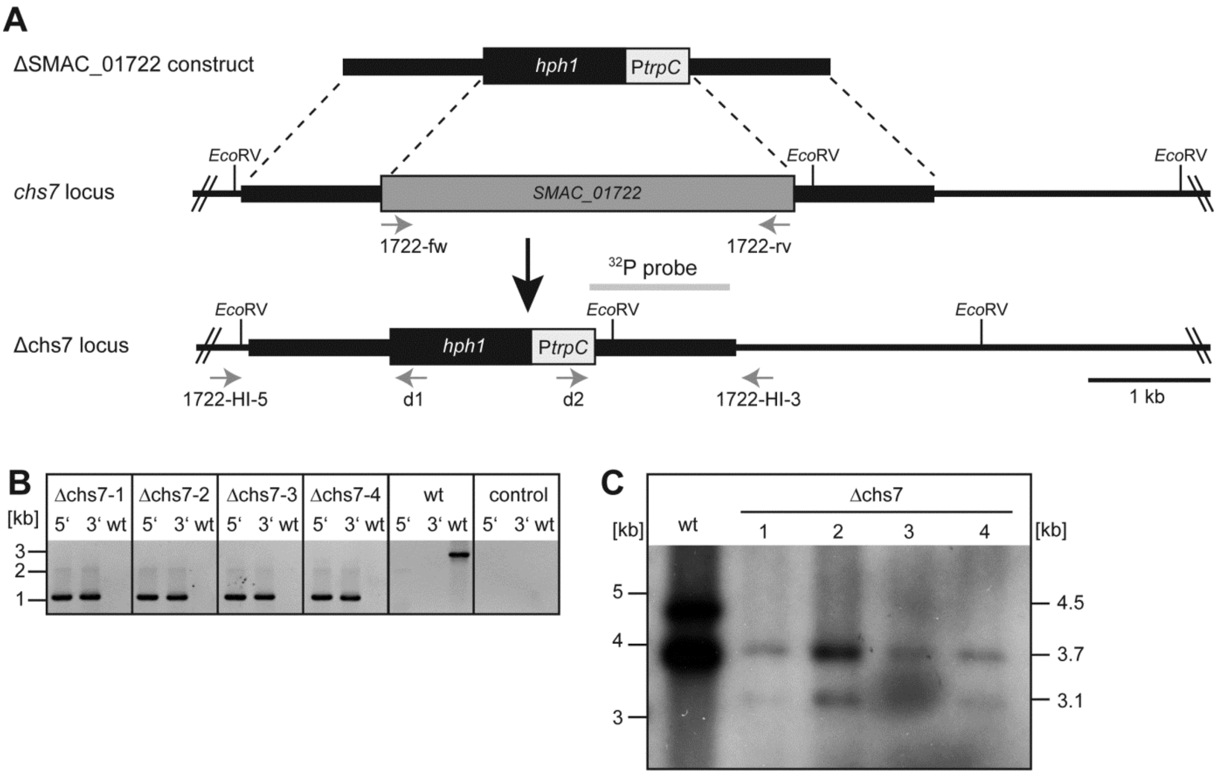

Figure S1. Deletion of *S. macrospora chs7*. **A.** Deletion strategy. Primers for verification are indicated by gray arrows, sequences are given in Table 2. **B.** PCR verification of *chs7* deletion strains. Primers used for amplification of the 5‘ (1722-HI-5/d1) and 3‘ (1722-HI-3/d2) flanks as well as the wild type *chs7* (1722-fw/1722-rv) are shown in A. **C.** Southern blot analysis of *chs7* deletion strains. Strains are the same as in B. Genomic DNA was digested with *Eco*RV and probed with the *chs7* 3‘ flank as indicated in A.

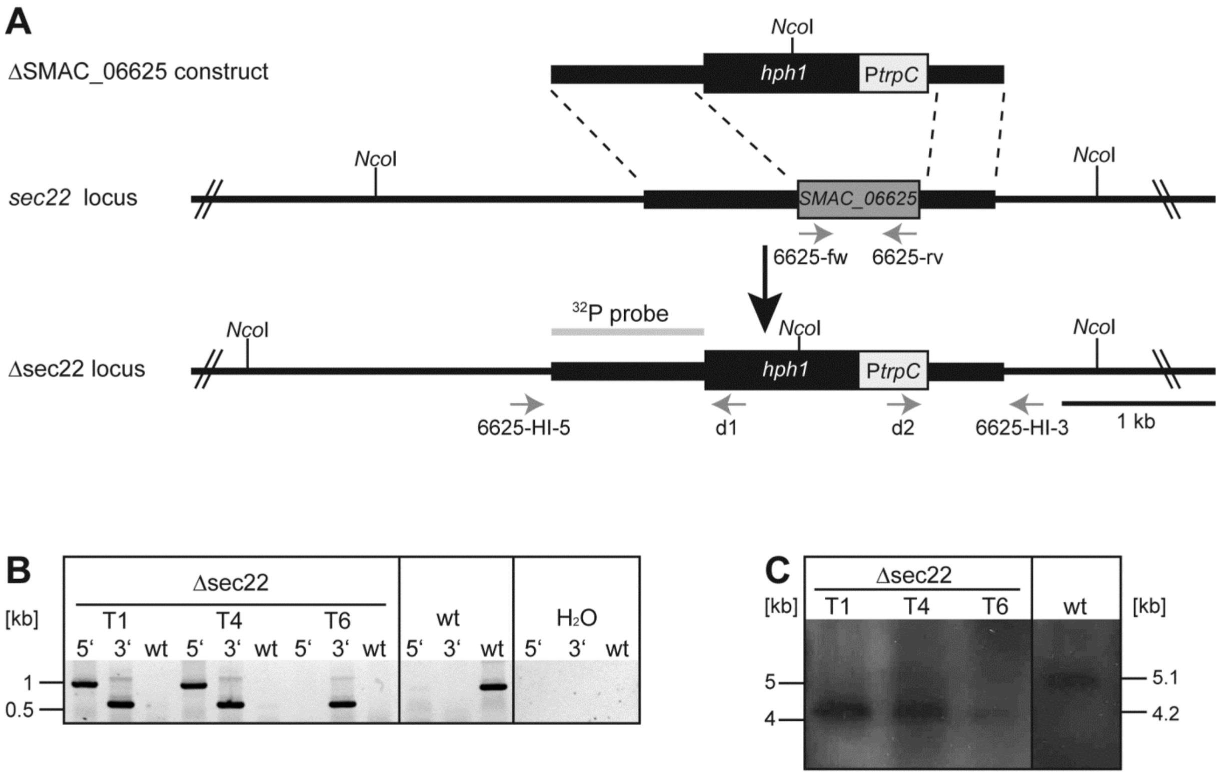

Figure S2. Deletion of *S. macrospora sec22*. **A.** Deletion strategy. Primers for verification are indicated by gray arrows, sequences are given in Table 2. **B.** PCR verification of *sec22* deletion strains. Isolate numbers of strains are S121285 (T1), S121345 (T4), and S121397 (T6). Primers used for amplification of the 5‘ (6625-HI-5/d1) and 3‘ (6625-HI-3/d2) flanks as well as the wild type *sec22* (6625-fw/6625-rv) are shown in A. **C.** Southern blot analysis of *sec22* deletion strains. Genomic DNA was digested with *Nco*I and probed with the *sec22* 5‘ flank as indicated in A.

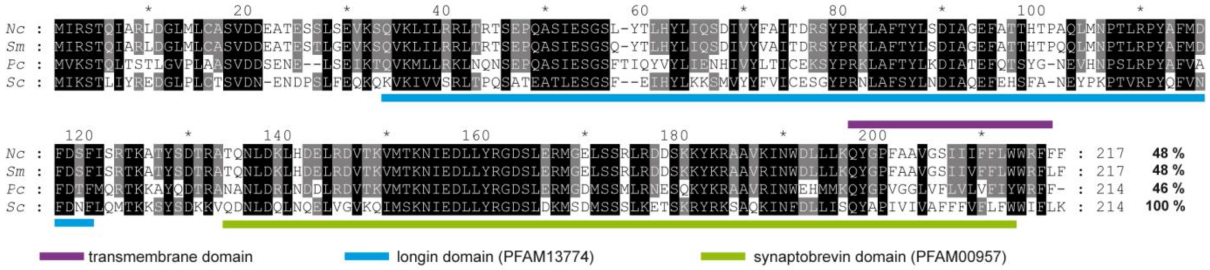

Figure S3. Multiple alignment of SEC22 homologs from four ascomycetes. SEC22 homologs from the Sordariomycetes *Neurospora crassa* (*Nc*, XP_960888.1) and *Sordaria macrospora* (*Sm*, SMAC_06625, XP_003346158.1), the Pezizomycete *Pyronema confluens* (*Pc*, CCX04245.1), and the Saccharomycete *Saccharomyces cerevisiae* (*Sc*, AAB67373.1) were aligned. Sequence identity compared to the *S. cerevisiae* Sec22p is given in % at the end of each sequence.

Table S1. Oligonucleotides used in the study.

Name	Sequence 5'-3'	Specificity
d1	CGATGGCTGTGTAGAAGTACTCGC	within *hph*
d2	ATCCGCCTGGACGACTAAACCAA	
426-3	CCCGATTTAGAGCTTGACGG	within pRS426 (nt 1651-1670)
426-5	AGCAGAGCGAGGTATGTAGG	within pRS426 (nt 2935-2954)
1722-fw	GACTATCAACTATCTGGGGG	detection of *chs7*
1722-rv	CTATCCCCTCGAACTCTGCC	
1722 5-fw	GTAACGCCAGGGTTTTCCCAGTCACGACGGGCAACATTACATTCTCCGG	amplification of 5' flank of *chs7* for knockout construct pΔSMAC_01722
1722 5-rv	CGAGGGCAAAGGAATAGGGTTCCGTTCGCTTGTCCCTTCTTTCTCC	
1722 3-fw	GCCCAAAAATGCTCCTTCAATATCAGTTGCGGACAGAGGTAGTTTGGG	amplification of 3' flank of *chs7* for knockout construct pΔSMAC_01722
1722 3-rv	GCGGATAACAATTTCACACAGGAAACAGACCAGTCACTTTGAGACGGACTTCA	
1722-HI-5	CGGCAGTATGTGCCTTTGGC	detection of homologous integration of *chs7* 5' flank
1722-HI-3	GGGGCTCCTATGACTATTG	detection of homologous integration of *chs7* 3' flank
6625-fw	CCGCTCAACACAAATTGCCC	detection of *sec22*
6625-rv	CAAAACAAGAACCTCCACCAC	
6625-5-fw	GTAACGCCAGGGTTTTCCCAGTCACGACG*GAATTC*CAGAGTTGCGCCGTAAAAGTCACCG	amplification of 5' flank of *sec22*for knockout construct pΔSMAC_06625
6625-5-rv	CGAGGGCAAAGGAATAGGGTTCCGTTGAGGTGGTGACGACCTTGGATTTCCTCG	
6625-3-fw	GCCCAAAAATGCTCCTTCAATATCAGTTGCGCGGATCATCACAGGCTGAGGCCAT	amplification of 3' flank of *sec22* for knockout construct pΔSMAC_06625
6625-3-rv	GCGGATAACAATTTCACACAGGAAACAGC*GAATTC*GCGGTTGCCATTTCTGTCTGGAACC	
6625-HI-5	CCGGGATTAGGTCCAAAATGCC	detection of homologous integration of *sec22* 5' flank
6625-HI-3	GGAAGTTGTGCGGTTATGTGATGG	detection of homologous integration of *sec22*3' flank
Prom6625-fw	CCCTCGAGGTCGACGGTATCGATAGGAACTATTCCCTTGTTCGCTCATCC	amplification of 1 kb upstream promotor region of *sec22*
Prom6625-rv	GCTTCACTTGCGACTTGACTTCGCC	
6625-gfp-fw	GGCGAAGTCAAGTCGCAAGTGAAGC	amplification of *sec22-egfp* fusion construct
6625-gfp-rv	ATGGCCTCAGCCTGTGATGATCCGCTTACTTGTACAGCTCGTCCATGCCG	
Term6625-fw	CGGCATGGACGAGCTGTACAAGTAAGCGGATCATCACAGGCTGAGGCCAT	amplification of 1 kb downstream terminator region of *sec22*
Term6625-rv	GCGGCCGCTCTAGAACTAGTGGATCATGAGTTCCAGCAACAGCAACAGCA	
SEC22-gfp-fw	ACAGCTACAGATCTAAGCTTATGATCCGCTCAACACAAAT	amplification of *sec22* with pDS23 overhang
SEC22-gfp-rv	CCTCGCCCTTGCTCACCATAAACAAGAACCTCCACCACAA	
SSU1	ATCCAAGGAAGGCAGCAGGC	RT-qPCR 18S rRNA
SSU2	TGGAGCTGGAATTACCGCG	
app-for2	GGAGATAGCTGGAGGGCTGA	RT-qPCR *app*
app-rev2	ATCTCGGGCTGACTTCCATC	
6625-qRT-fw	AGGGACGTCACCAAGGTCAT	RT-qPCR *sec22*
6625-qRT-rv	CATCCCTCAACCGACTGCT	

1930-qRT-fw	GGTGAAAAGCTTGACGACCTG	RT-qPCR *ykt6*
1930-qRT-rv	CCAGACAGCACGAGTTTTGC	
ppg1-for	CTCCGTGACACCACCTTCAG	RT-qPCR *ppg1*
ppg1-rev	GGAGGCATAGCGCTTCCA	
ppg2for	CGGTATCTCGCCTCTCAACGT	RT-qPCR *ppg2*
ppg2rev	GTTGTGCTCCCATTGTGCAGA	
Smta-1-for	TGATCCGCACTCACTTCCAT	RT-qPCR *Smta-1*
Smta-1-rev	GGGAGTGGCATCAACCGTAT	
SmtA-2-for	TCGCCATGACAGCATCTTCT	RT-qPCR *SmtA-2*
SmtA-2-rev	GTCGAGCGAAAACCTTGGAG	
nox1_RT_fw	GGACATGGATACCACGCAGA	RT-qPCR *nox1*
nox1_RT_rv	TTCCGCATGCTCTCAAAGAA	
nox2_RT_fw_2	CTGGTTCTTTTCCCCGTCTG	RT-qPCR *nox2*
nox2_RT_rv_2	GGACCATGCTGTCGTGATGT	
sac1_RT_fw	AGGCTTGCACTTCTCTTCGG	RT-qPCR *sac1*
sac1_RT_rv	TTGAGCAGGCCCGTTAATCT	
SMU3584for	GGTCATGGGCCACAGTCTCG	RT-qPCR *pks*
SMU3584rev	CGTGGCTGTTCATCGTGCAC	
SMU6905for	GGCATCACGGTCAATGGTGT	RT-qPCR *teh*
SMU6905rev	TGCTCAGCCATCATCCTCTCA	
SMU9390for	TCAACATCAACACCCGTGGC	RT-qPCR *tih*
SMU9390rev	GTAAACAGCGTGCTTGGGCA	
pro41-for	ACATGGAGGCAAATGGGAAG	RT-qPCR *pro41*
pro41-rev	CGTCTGAGCCAATGATGCTC	
