# Supplementary material for: Functional Analysis of Developmentally Regulated Genes chs7 and sec22 in the Ascomycete Sordaria macrospora
Source: G3 (Bethesda). 2015 Apr 14;5(6):1233–45. doi: 10.1534/g3.115.017681 (PMC4478551; doi:10.1534/g3.115.017681)
Supplement: Supporting Information [file supp_g3.115.017681_FigureS1.pdf]

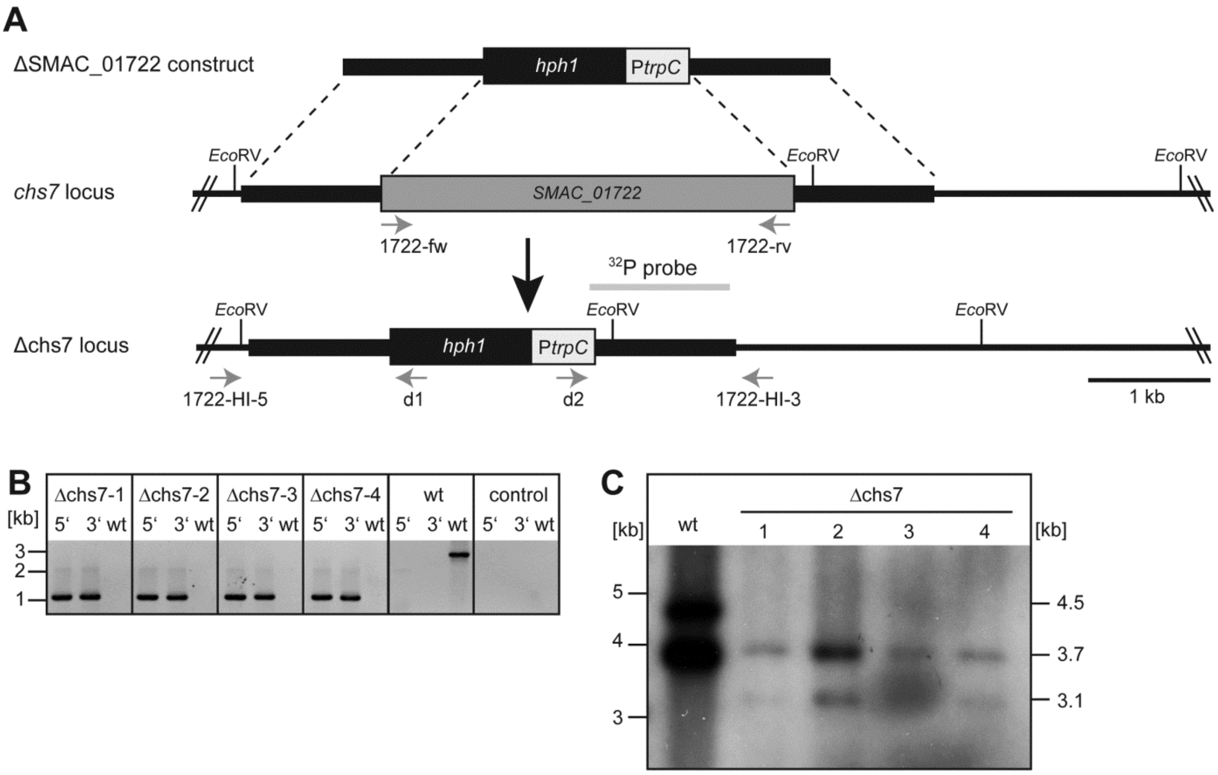

Figure S1. Deletion of *S. macrospora chs7*. **A.** Deletion strategy. Primers for verification are indicated by gray arrows, sequences are given in Table 2. **B.** PCR verification of *chs7* deletion strains. Primers used for amplification of the 5‘ (1722-HI-5/d1) and 3‘ (1722-HI-3/d2) flanks as well as the wild type *chs7* (1722-fw/1722-rv) are shown in A. **C.** Southern blot analysis of *chs7* deletion strains. Strains are the same as in B. Genomic DNA was digested with *Eco*RV and probed with the *chs7* 3‘ flank as indicated in A.
