# Supplementary material for: Functional Analysis of Developmentally Regulated Genes chs7 and sec22 in the Ascomycete Sordaria macrospora
Source: G3 (Bethesda). 2015 Apr 14;5(6):1233–45. doi: 10.1534/g3.115.017681 (PMC4478551; doi:10.1534/g3.115.017681)
Supplement: Supporting Information [file supp_g3.115.017681_FigureS2.pdf]

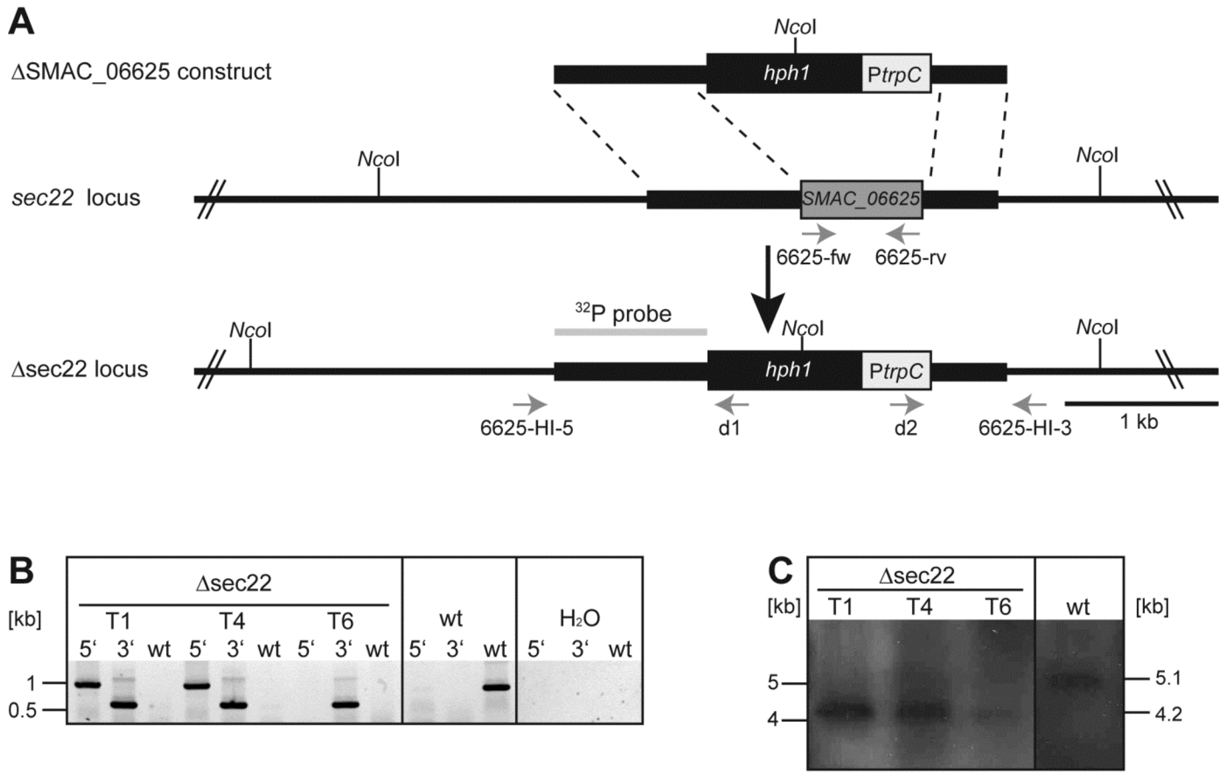

Figure S2. Deletion of *S. macrospora sec22*. **A.** Deletion strategy. Primers for verification are indicated by gray arrows, sequences are given in Table 2. **B.** PCR verification of *sec22* deletion strains. Isolate numbers of strains are S121285 (T1), S121345 (T4), and S121397 (T6). Primers used for amplification of the 5' (6625-HI-5/d1) and 3' (6625-HI-3/d2) flanks as well as the wild type *sec22* (6625-fw/6625-rv) are shown in A. **C.** Southern blot analysis of *sec22* deletion strains. Genomic DNA was digested with *Nco*I and probed with the *sec22* 5' flank as indicated in A.
