# Supplementary material for: Functional Analysis of Developmentally Regulated Genes chs7 and sec22 in the Ascomycete Sordaria macrospora
Source: G3 (Bethesda). 2015 Apr 14;5(6):1233–45. doi: 10.1534/g3.115.017681 (PMC4478551; doi:10.1534/g3.115.017681)
Supplement: Supporting Information [file supp_g3.115.017681_FigureS3.pdf]

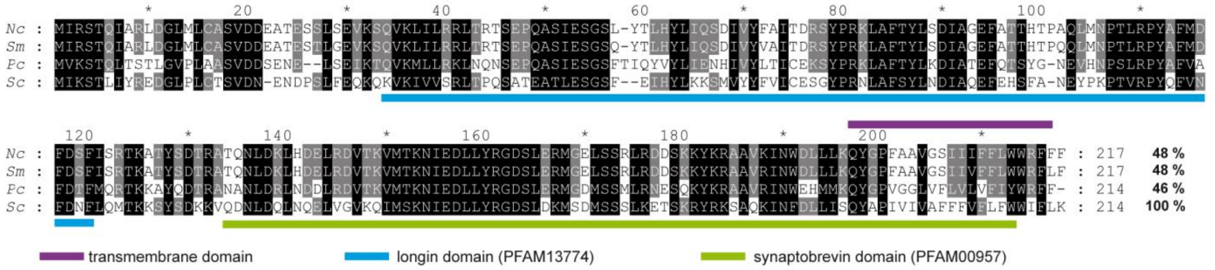

Figure S3. Multiple alignment of SEC22 homologs from four ascomycetes. SEC22 homologs from the Sordariomycetes *Neurospora crassa* (*Nc*, XP_960888.1) and *Sordaria macrospora* (*Sm*, SMAC_06625, XP_003346158.1), the Pezizomycete *Pyronema confluens* (*Pc*, CCX04245.1), and the Saccharomycete *Saccharomyces cerevisiae* (*Sc*, AAB67373.1) were aligned. Sequence identity compared to the *S. cerevisiae* Sec22p is given in % at the end of each sequence.
